# Gold-iron oxide nanohybrids: insights into colloidal stability and surface-enhanced Raman detection†

**DOI:** 10.1039/d1na00455g

**Published:** 2021-09-08

**Authors:** Sebastian P. Schwaminger, David Bauer, Paula Fraga-García

**Affiliations:** Bioseparation Engineering Group, Department of Mechanical Engineering, Technical University of Munich Boltzmannstr. 15 Garching Germany s.schwaminger@tum.de p.fraga@tum.de

## Abstract

Nanoparticles are acquiring an ever increasing role in analytical technologies for enhanced applications such as signalling of hazardous dyes. One challenge for the synthesis of hybrid nanomaterials is to control their shape, size and properties. The colloidal and interfacial properties of initial nanoparticles are decisive for the formation, growth and characteristics of nanohybrids. Our objective is to combine the advantages of iron oxide nanoparticles for magnetic separation with nanoscale gold for a surface enhanced Raman scattering (SERS) effect which could be used *e.g.* for improved detection of dye molecules. We synthesized iron oxide nanoparticles (∼10 nm) with a high saturation magnetization of around 80 Am^2^ kg^−1^ and coupled nanoscale gold to these particles. The focus was set in testing multiple approaches to combine these two materials with the goal of understanding and discussing the effect of the colloidal stability of iron oxide nanoparticles on the properties of the hybrid material. Stability is a seldom addressed issue; however, it plays a critical role for guaranteeing a homogeneous distribution of the gold on the iron oxide surface. We characterized the produced materials with UV/Vis spectroscopy, dynamic light scattering, and transmission electron microscopy, and their capability to enhance Raman signals is investigated. The seed-mediated growth method of oleate and PEG-stabilized magnetic particles yielded the best enhancement of Raman scattering for identification of the dye Rhodamin 6G. This approach can be used to couple gold nanoparticles to other surfaces and microfluidic devices. The presented method might pave the way to further applications in diagnostics or also in environmental approaches and beyond.

## Abstract 2

Colloidal stability affects multiple properties at the nanoscale. Not only the synthesis of hybrid gold-iron oxide nanoparticles but also their ability to enhance Raman signals (SERS) is determined by their surface modification.

## Graphical abstract

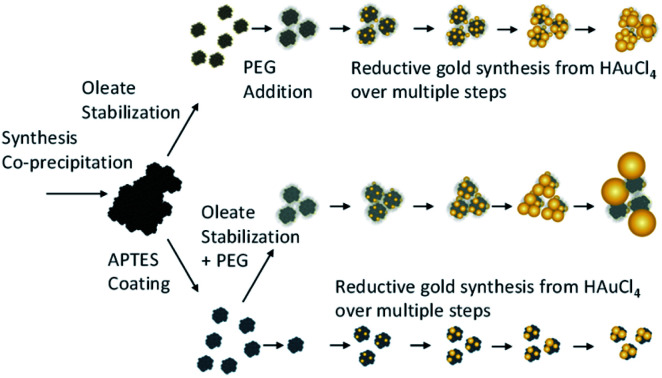

## Introduction

Gold has been a fascinating attraction for humans since ancient times. In the nanoscale, gold reveals its full potential due to the high surface-to-volume ratio. From the application in catalytic CO_2_ reduction to sensing properties, gold nanoparticles outshine many other materials.^1,2^ The tuning of the size and shape allows a plasmon frequency in the range of the visible light and therefore gold nanoparticles can adopt any color.^2–4^ Due to this effect, gold nanoparticles have been used for coloring glass for centuries.^4,5^ Furthermore, the specific plasmon frequency of nanoscale gold allows an enhancement of Raman scattering.^5–8^ Localized surface plasmons of gold electromagnetically interact with their surroundings and increase the probability of the otherwise rare Smekal-Raman effect (1 : 10^10^).^9–13^ This effect has been first observed by Fleischmann *et al.* in 1974 on a silver electrode.^14^ Since this time, multiple sensing applications have been derived from the surface-enhanced Raman (SERS) effect.^15–18^ Particularly gold particles have been in the focus of interest due to their superior stability compared to other noble metal nanoparticles such as silver or copper.^19^ The SERS effect, which is caused by these noble metal nanomaterials, can be used to significantly improve the limit of detection for multiple molecules.^20^ This is of special interest for molecules that need to be identified already at very low concentrations. SERS can be applied in pharmaceutical diagnostics^21^ as well as for the detection of explosives^22^ or in forensic science.^23^ However, scarcity makes gold an expensive material. Therefore, this precious material is rarely used in its bulk form, but rather as a thin coating on other materials, *i.e.*, in core–shell structures. This usage permits benefitting from a quantum size effect from the advantageous gold properties while needing only a small amount of noble metal.

Other materials often used for sensing applications, due to their amazing properties at the nanoscale, are magnetic nanoparticles.^24^ Due to the confinement of iron ions at the nanoscale, the magnetic spins of their electrons tend to rotate depending on the temperature, providing superparamagnetic properties to the nanoparticles.^25^ This superparamagnetism allows manipulation of magnetic nanoparticles in a magnetic field while the particles show no remanent magnetization. This is the basis for magnetic separation approaches where molecules, bound to magnetic adsorbents, can be magnetically separated using a magnet and thus can be concentrated.^26,27^ Multiple materials can be used as magnetic carrier adsorbents.^28^ Here iron, cobalt or nickel as well as lanthanides or metal oxide particles are typically used.^28^ The iron oxides magnetite and maghemite represent a low-cost class of these materials. Their properties can be controlled by synthesis to achieve high saturation magnetizations and zero magnetic remanence.^29^ A plethora of synthesis techniques with advantages and disadvantages exist: co-precipitation,^29^ sol–gel,^30^ microemulsion,^31^ solvothermal,^32^ hydrothermal,^33^ sonochemical^34^ and multiple other routes.^35^

Since iron oxide and gold nanoparticles show these beneficial and amplifying properties for molecule detection, many approaches exist to combine both particle types.^18,35–37^ Typical approaches are the synthesis of Janus or dumbbell particles or the coating of iron oxide nanoparticles with gold nanoparticles as core–shell composites, flowers, stars, *etc.*^36^ Both the synthesis route and the presence of stabilizing agents influence the shape and properties of bifunctional iron oxide-gold nanoparticles.^38–40^ The most widespread synthesis route is based on the epitaxial growth of the individual components in a seed-mediated process.^25^ Here, the attachment of small or large gold particles to iron oxide nanoparticles in the form of Janus particles, complete gold shells and spiky gold structures can be created.^18,36,39,41^ The arrangement and the form of the plasmonic gold nanostructures on the magnetic nanoparticles define the enhancement effect and therefore the intensity of the SERS signal.^41^ Of special interest is the use of gold on magnetite and maghemite nanoparticles, which permits fusing superparamagnetic properties with the high stability, the particularly rich surface chemistry and the enhanced optical properties of gold.^42^ Here, two important question arise: How can one optimize the interplay of both materials; and more particularly, what is the best strategy to synthesize particles which show an improved SERS effect and can be easily manipulated in magnetic fields? This work is motivated by these questions and the aim of finding a successful, but simple and fast, synthesis route. We present multiple approaches for the attachment and growth of nanoscale gold on magnetic nanoparticles (MNPs). The controlled coating of MNPs with gold is an extremely challenging task, as pointed out by Wu *et al.*^42^ We discuss the critical role of the colloidal stability of MNPs for guaranteeing a homogeneous distribution of gold seeds on the SPION surface and for diminishing the probability of non-homogeneous growth (epitaxial or cascadal). Therefore, we characterize the particles based on their optical, magnetic and SERS properties. A large issue for the SERS effect is its stochastic instability, and therefore multiple synthesis approaches towards Raman spectroscopy are necessary to verify the properties of the synthesized materials.

## Experimental

### Synthesis of magnetite nanoparticles

Sodium hydroxide (1.12 mol, 44.789 g) was dissolved in 500 mL of degassed, deionized water and cooled to 25 °C in a laboratory stirring reactor (OptiMax, Mettler Toledo, Germany) under a nitrogen flow. A 200 mL solution containing 0.20 mol FeCl_3_·6H_2_O (54.058 g) and 0.1 mol FeCl_2_·4H_2_O (19.883 g) was added to the base at a stirring velocity of 900 rpm for 30 min. Magnetic decanting and washing were performed until the conductance of the suspension dropped below 200 μS cm^−1^. The final suspension was stored in a sealed glass bottle under a nitrogen atmosphere. The same reactor was used for all syntheses.

### Synthesis of oleate and PEG-stabilized gold-MNP hybrids

To obtain oleate coated particles, a suspension containing 60 mg MNPs (1 g L^−1^) was mixed with a 12.0 mL solution of 25 vol% ammonia and sonicated (Branson Sonifier) for two minutes. To the mixture 30.0 mL of oleate (1 g L^−1^) were added, mixed and sonicated (1 min). The particles were washed twice with 60 mL of deionized water. The particles were mixed with 15 mL of a PEG4000 (10 g L^−1^) solution and incubated for 24 h at room temperature. The suspension was washed two times with deionized water. The particles were adjusted to a concentration of 0.1 g L^−1^ and a pH of 12 with NaOH. The mixture was sonicated prior to the addition of sodium citrate (3 mL, 0.1 M) and stirred for 5 min. 3 mL hydroxylamine hydrochloride NH_2_OH(HCl) (0.2 M) and 6.5 mL HAuCl_4_ (29 mM) were added successively in 5 iteration steps. After each step, the UV/Vis spectrum was measured. After the last iteration the mixture was kept under stirring for another 30 minutes, before washing it three times with deionized water.

### Synthesis of amine-functionalized gold-MNP hybrids

To 500 mL of a suspension containing 250 mg of freshly prepared Fe_3_O_4_ nanoparticles, 10 mL of a glycine/NaOH buffer (0.2 M Gly, 0.2 M NaCl, 0.1 M NaOH) were added to achieve a pH of 9.45. The mixture was sonicated for 10 min and stirred at 600 rpm in a laboratory reactor (OptiMax). After addition of 0.625 mL of 3-aminopropyltriethoxysilane (APTES) (0.59 g, 2.7 mmol) dissolved in 50 mL ethanol (96.4 vol%), the mixture was stirred for 3 hours and then washed three times with deionized water. The particles were treated again with glycine buffer (0.2 M Gly, 0.2 M NaCl, 0.1 M NaOH) before using them in coating experiments. Therefore, 6 mL buffer were mixed with 18 mL MNP-APTES suspension (2.5 g L^−1^) and sonicated (1.5 min). The gold coating was similar to the synthesis described above. The only differences were a lower amount of citrate (2 mL), a higher amount of NH_2_OH (3.75 mL) and a lower amount of HAuCl_4_ (5.27 mL) added to the suspension.

### Synthesis of amine-functionalized and oleate- and PEG-stabilized gold-MNP hybrids

Both synthesis routes, which have been described above, have been combined. The particles were first coated with APTES and afterwards stabilized with oleate and PEG following the same procedures. For the gold coating the same route as for the stabilized MNPs was followed. All synthesis conditions are listed in Table S1†.

### Characterization

Dynamic light scattering (DLS) and zeta potential measurements were conducted with a Delsa Nano C Particle Analyzer (Beckman Coulter, Germany). The CONTIN method was used to conduct 100 DLS measurements for each sample. For the pH adjustments, HCl and NaOH (0.1 M) were used as titrants. Infrared spectra were recorded with a Bruker Vertex 70 (Bruker Optics GmbH, Ettlingen, Germany), using a Platinum attenuated total reflection (ATR) unit and a mercury cadmium telluride detector. Therefore, samples were freeze-dried with a LPHA1-2 LDplus lyophilizer and placed on the sample plate of the spectrometer and measured with 32 scans in a wave number range from 400 to 4000 cm^−1^.

A Senterra Raman spectrometer (Bruker Optics GmbH, Ettlingen, Germany), equipped with a 785 nm and 488 nm laser, was used for SERS measurements as well as an A Lab-RAM One (Horiba) Raman spectrometer with a He–Ne gas laser of 633 nm laser. For SERS measurements, 50 μL of the sample were placed in the mold of a single molded microscope slide for all experiments. For transmission electron microscopy (TEM), a JEM-1400F (JEOL Ltd., Japan) microscope was used. Colloidal samples were diluted in deionized water, and sonicated and a droplet of each sample was placed on a carbon coated copper grid (Quantifoil Micro Tools GmbH, Germany). The grids were allowed to dry under ambient conditions. Each copper grid was analysed with a number of two or three pictures from different locations. The pictures were manually processed in ImageJ. A number of at least 150 particles per sample was measured in a randomized order. Only particles from the edges of the precipitated aggregates were counted where a clear differentiation between the particles was possible. For photometric measurements in this study the photometer Infinite 200 Pro (Tecan Trading AG, Switzerland) was used for UV/Vis spectroscopy. The samples were measured in UV-microtiter plates (Nunc, Thermo Fisher Scientific Inc., US). Therefore, 200 μL of the sample were used. If not described differently, spectra between 230 and 850 nm wavelengths were recorded in 5 nm steps.

## Results and discussion

Iron oxide nanoparticles were prepared by a co-precipitation route which yielded magnetic nanoparticles with a high saturation magnetization of 82 A m^2^ kg^−1^ and no remanence magnetization at 300 K.^43^ The diameter of these nanoparticles obtained with transmission electron microscopy ranges from 5–18 nm with an average value of approx. 10 nm.^43^ The nanoparticles demonstrate the common amphoteric characteristics with a positive zeta potential at acidic pH and a negative zeta potential at basic pH (Fig. S1†).^43^

### Hydrodynamic properties and colloidal stability

While the primary particle diameter is in the nanoscale, the particles agglomerate to μm-sized units especially at neutral pH, where the zeta potential is close to zero and therefore electrostatic repulsion is only moderate.^44^ This aggregation behavior can be beneficial for separation processes.^44,45^ However, to sythesize gold-coated hybrid nanoparticles, the aggregation of iron oxide nanoparticles is a challenge.^18,36^ For a homogeneous gold coating, stable nanoscale materials are needed.^46,47^ Hence, we evaluated different stabilization methods for the gold coating in this work. We want to emphasize that synthesizing gold-coated particles that enhance the Raman signals of molecules while allowing magnetic separation is still challenging.^18^ Therefore, we want to compare different relatively simple approaches of stabilizing iron oxide nanoparticles focusing on wet-chemical methods at ambient temperature using non-toxic precursors. On the one hand, iron oxide nanoparticles were stabilized with a silane coating with APTES, yielding positively charged amine surface groups (Fig. S1†).^48^ On the other hand, iron oxide nanoparticles were stabilized with oleate. Oleate stabilization leads to a lipid bilayer formation around the nanoparticles and therefore to steric and electrostatic stabilization.^49,50^ The electrostatic stabilization through the carboxy group which protrudes out of the lipid bilayer leads to a negatively charged surface at neutral pH and therefore repulsion between nanoparticles.^49^

**Fig. 1:**
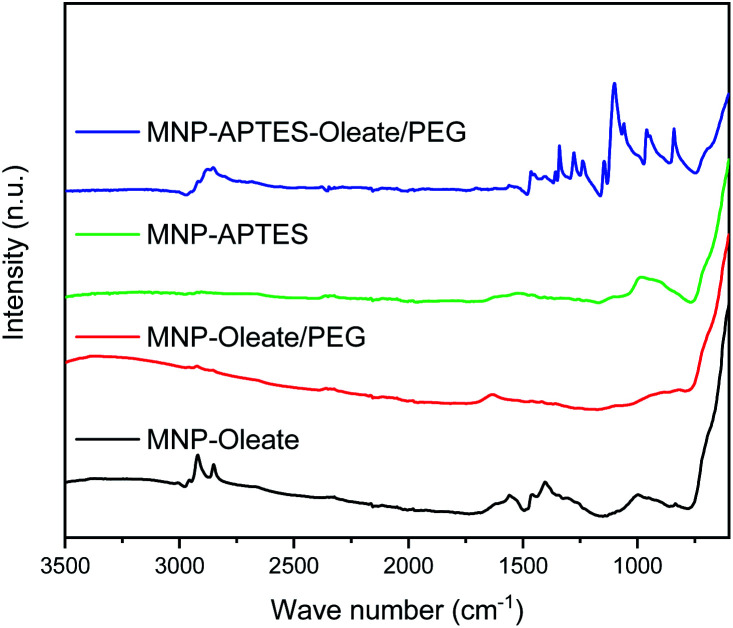
ATR-IR spectra of dried differently coated MNPs.

**Fig. 2:**
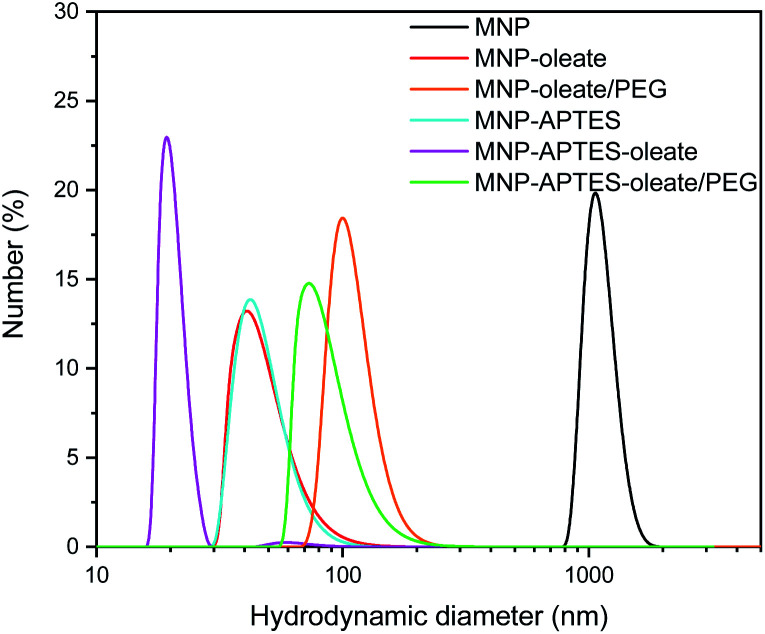
Hydrodynamic diameters of MNPs and stabilized MNPs showing particle aggregates as obtained with DLS at pH 7.

The oleate molecules are anchored *via* a bidentate binding state, which can be observed with FTIR spectroscopy (Fig. 1).^49,50^ Here, multiple peaks around 1558 and 1423 cm^−1^ corresponding to the asymmetric and symmetric stretching vibrations of COO^−^, respectively, can be observed.^49,51^ Furthermore, the C–H stretching vibrations of oleate are visible at 2960 and 2870 cm^−1^.^51^ The thickness of the lipid bilayer, which is up to 4 nm, inhibits magnetic dipole interactions between the iron oxide nanoparticles.^50^ The coating with oleate leads to a significant deagglomeration of the nanoparticles and a decrease of the hydrodynamic diameter from 1000 nm to 45 nm, which is in good agreement with the literature (Fig. 2).^50^ Even when stabilized, the nanoparticles do not occur as single nanoparticles but as nanoaggregates consisting of primary nanoparticles.

Hence, the oleate coating is a good approach to increase the colloidal stability of iron oxide nanoparticles. However, a lipid bilayer is not rigid. Lipid bilayers are known to diffuse and molecules can exchange locations.^52^ Particularly when the suspension is mixed with another aqueous system, the lipid bilayer redistributes itself due to diffusion. The growth and inclusion of multiple particles within the bilayers can also not be excluded. The system reorganizes and reshapes itself over time. Consequently, a more rigid coating seems to present itself, as we demonstrate below.

The silanization of APTES on iron oxide nanoparticles yields electrostatically stabilized nanoparticles as well and deagglomeration can be observed to the same extent as for oleate-coated nanoparticles.^48^ The hydrodynamic diameter of the APTES-coated particles is around 45 nm as well (Fig. 2). A successful coating can be observed in the FTIR spectrum, where Si–O–Si and Fe–O–Si vibrations are visible at 1111, 1049 and 1018 cm^−1^.^48,53^ Furthermore, the bending vibration of N–H is visible at 1500 cm^−1^.^53^ The successful coating with APTES can further be observed with thermogravimetric analysis.^54^ Here, the APTES-coated particles demonstrate multiple thermal degradation steps leading to a total mass loss of 14% compared to 4% of bare iron oxide nanoparticles (Fig. S2†).

In addition to the coating with APTES, a further stabilization of the APTES-coated nanoparticles with oleate is tested. This combination yields the best particle stabilization and hydrodynamic diameters of around 20 nm can be reached; this value corresponds to the size of single coated nanoparticles (Fig. 2). The oleate coating can be verified by FTIR spectroscopy: C–H and COO^−^ vibrations appear between 2900 and 3000 cm^−1^ and between 1600 and 1400 cm^−1^, respectively (Fig. 1).^49^.

A further hydrophilic stabilizer material that was tested is polyethylene glycol (PEG). Its properties in stabilizing nanoparticles and lipid bilayers are beneficial to the synthesis and coating with gold and therefore PEG was investigated.^55^ Due to their chain length, the PEG molecules lead to a larger hydrodynamic diameter, even though the nanoparticles are stabilized by them.^40,48^ Here, PEG leads to hydrodynamic diameters of around 100 nm for oleate coated nanoparticles and to 80 nm for APTES and oleate-coated nanoparticles (Fig. 2). The PEG coating is clearly visible in the FTIR spectra of APTES and oleate-coated nanoparticles (Fig. 1).^55^ Particularly the C–O–C vibration at 1100 cm^−1^ is very prominent.^48,55^ However, for the oleate coated nanoparticles only a slight increase in absorbance between 700 and 1100 cm^−1^ is visible when PEG is added.

### Seed-mediated growth of gold particles on MNPs

**Fig. 3:**
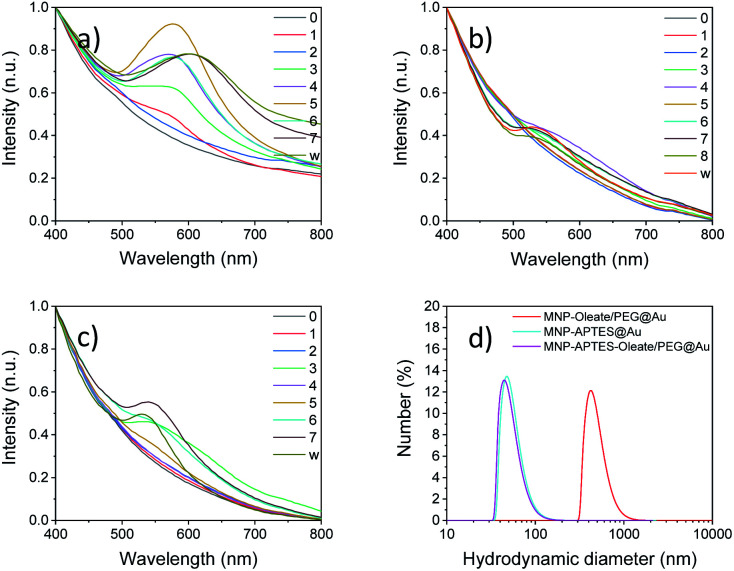
UV/Vis spectra for multiple steps in the gold coating process for (a) MNP-oleate/PEG, (b) MNP-APTES and (c) MNP-APTES-oleate/PEG. (d) Hydrodynamic diameter showing particle aggregates of all three products as measured with dynamic light scattering.

The stabilized particles are incubated with a reductive agent and HAuCl_4_, which is a typical precursor for the synthesis of gold nanoparticles.^39^ Over 7 or 8 iterative reaction steps, depending on the synthesis, the change of absorbance in the visible light is detected. Therefore, the region between 400 and 800 nm is scanned with a UV/Vis spectrometer. For the oleate- and PEG-stabilized nanoparticles, the first change of the system is visible after two coating procedures with a small absorbance band below 560 nm (Fig. 3a). This is an indicator of the formation of gold particles that are smaller than 70 nm.^56^ With every synthesis step, this band increases and slightly shifts to higher wavelengths. This changing absorbance can be observed from the UV spectra in Fig. 3a as well as with the naked eye (Fig. S3†). A maximum is reached after 5 and 6 reactions at around 590 nm. This is in good agreement with the literature on similar synthesis approaches^57^ and with the absorbance of bare gold nanoparticles^58^ (Fig. S4 and S5†).^46,59^ The route based on maghemite nanoparticles and seeds for reductive gold synthesis follows a similar trend of UV absorbance spectra to the oleate-stabilized nanoparticles (Fig. 3a and S4†). However, the maximum of the tetramethyl ammonium-stabilized particles is a little bit blue-shifted at 570 nm. The seventh reaction leads to a significant red shift of the gold coating to an absorbance maximum at 610 nm. This wavelength cannot solely be explained by the gold content but by the interaction of gold and iron oxide nanoparticles.^46,59^ The magnetic separation and washing of the nanoparticles do not affect the absorption of the particles, which is a good indicator of the stable coating of iron oxide nanoparticles with a gold layer.^46^

The nanoparticles stabilized with APTES, with the smallest hydrodynamic diameter of all nanoparticle systems investigated, demonstrate a similar behavior of increasing absorbance of visible light with reaction steps. However, significant changes in the absorbance behavior can only be observed after 4–5 reaction steps (Fig. 3b). The observable band at around 550 nm is significantly blue-shifted compared to the oleate- and PEG-stabilized systems, which is an indicator of smaller or thinner gold layers.^55^ However, even with the following reactions, the band does not increase in size significantly and shift towards an absorbance maximum around 600 nm. The nanoparticles stabilized with APTES, oleate and PEG show a mixed behavior compared to the other two synthesis routes (Fig. 3c). Here, bands are visible after 3 reaction steps and the absorbance at around 550 nm is less pronounced and slightly blue-shifted compared to the particles shown in Fig. 3a. This means that the size of the gold nanoparticles is smaller compared to that observed in the first synthesis. This is in good agreement with the stabilization and the smaller hydrodynamic diameter of this system.^36^ Not only the initial but also the final hydrodynamic diameter of the oleate- and PEG-stabilized nanoparticles is significantly larger compared to those of APTES-stabilized nanoparticles (Fig. 3d). The oleate- and PEG-stabilized particles yield gold-coated particles with a hydrodynamic diameter of around 500 nm. Both other gold coatings lead to smaller hydrodynamic diameters which are around 50 nm (Fig. 3d). This aggregation behavior might also explain a different UV/Vis background for the PEG and oleate stabilized iron oxide particles coated with gold particles. For the UV/Vis spectra of the particles, a higher absorbance can be observed at 700–800 nm compared to the other spectra in Fig. 3. Therefore, the gold coating affects the hydrodynamic diameter significantly, probably due to the different kinetics of seeded growth as a consequence of an inhomogeneous distribution of the seeds depending on the process conditions.^57^ A similar stabilization behavior can be observed for gold-coated maghemite nanoparticles as described in the ESI where a hydrodynamic diameter of 96 nm can be observed after 10 seeding steps with HAuCl_4_ in the presence of hydroxylamine, based on the procedure of Lyon *et al.* (Table S1†).^57^

**Fig. 4:**
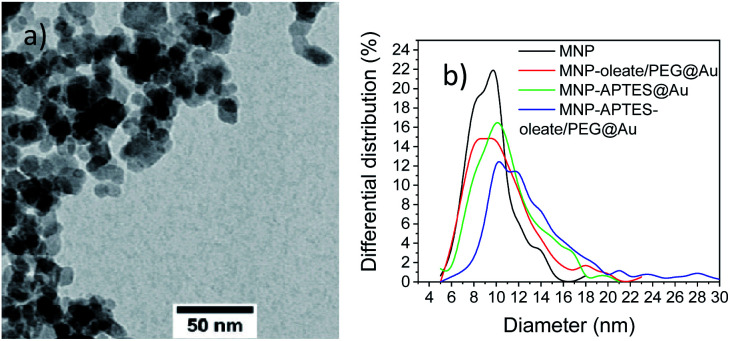
(a) TEM microscopy image of MNP-APTES-oleate/PEG@Au particles and (b) differential size distribution of the primary particle sizes from TEM analysis of all synthesized gold-coated particles and bare MNPs.

In a very recent review, Lavorato *et al.* explained the use of seed-mediated approaches for hybrid magnetic nanoparticles.^25^ They state the importance of the difference in the surface energy between the seeds and the nuclei; when this energy is large, heterogeneous nucleation may be blocked due to the high energetic barrier, which can lead to asymmetry in the growth of the second phase and the development of anisotropic hybrid structures.^25^ These hybrid structures can be observed often for materials with similar crystal structures. However, the affinity of the crystallizing material to the seeding material is another decisive factor for hybrid shapes and properties.^25^ Here, not only the colloidal stability and the heterogeneity of the MNP seeds but the nature of surfactants affect the growth of gold nanoparticles.^18^ The diameter observed with TEM microscopy (Fig. 4) significantly diverges from the hydrodynamic diameter. This observation strongly correlates with the tendency of small nanoparticles to aggregate and therefore even stabilized and small aggregates are visible in the DLS. On the other hand, even in TEM aggregates are visible and only the primary particles forming these aggregates are considered for counting and measuring. While transmission electron microscopy is a great technique to visualise the size and shape of nanoparticles, the nanoparticles cannot be imaged in their native dispersion state but precipitated on a carbon film which affects the aggregates and might even form new and larger aggregates on the surface. The different preparation and measurement technique orthogonally add up on how the nanoparticle system behaves but each measurement technique is not able to visualise all aspects of such complex systems on the nanoscale. The bare MNP demonstrates a diameter of around 10 nm with a narrow size distribution.^43^ APTES coating and gold coating lead to an increase in particle sizes of nanoparticles (Fig. 4). The increase of the particle diameters observed in TEM images is in good agreement with the literature.^46^ However, small nanoparticles can still be observed in the TEM micrographs. The nanoparticles based on oleate, and PEG stabilization yield an average particle diameter of 11 nm and show larger particles than bare MNPs. This behavior indicates a very thin coating or the attachment of small gold nanoparticles to the iron oxide nanoparticles (Fig. 4b and S6a†).^46^ The APTES-coated nanoparticles also possess an average diameter of around 11 nm, which indicates a similar behavior to the oleate-coated nanoparticles (Fig. 4b, S6b and c†). However, the APTES coating itself already contributes to an increase in particle size and therefore, the gold coating might even be thinner and the particles smaller.^53^ The APTES-, oleate- and PEG-stabilized nanoparticles yield the largest gold nanoparticles with a shift of the average particle size to 13 nm (Fig. 4). However, multiple significantly larger particles can be observed in the TEM images as well (Fig. 4). As a comparison, the TMAOH-stabilized maghemite particles behave similarly and show hetereogeneous gold particles, which are slightly larger than the iron oxide nanoparticles (Fig. S6d†). The synthesized bare gold nanoparticles are slightly larger (30–40 nm) which can be observed with scanning electron microscopy in comparison to the hybrid nanomaterials (Fig. S7†). The observations with UV/Vis spectroscopy and TEM can be further verified with XRD analysis (Fig. S8†). For all gold-coated nanoparticles the pattern of gold is most prominent even though cubic iron oxide is visible as well in the form of the 311 reflection of magnetite and maghemite. The gold 111 reflection is clearly visible in all patterns and indicates the presence of nanoscale gold. The broading of the 111 reflection yields the smallest gold particles for the APTES-coated particles (17 nm) and the largest gold particles for APTES-coated, oleate- and PEG-stabilized nanoparticles (41 nm). The oleate- and PEG-stabilized MNPs demonstrate a gold nanoparticle diameter of 32 nm, which is derived with the Scherrer equation (Fig. S8†). The TMAOH-stabilized maghemite nanoparticles also indicate the presence of maghemite in their diffractogram even though the reflections derived from gold are much more intense. Scherrer analysis yields a diameter of 27 nm (Fig. S9†). The X-ray analysis only considers gold nanomaterials, which explains the significant discrepancy between the TEM and the Scherrer diameter (Table S1†).

### SERS studies of hybrid nanoparticles

**Fig. 5:**
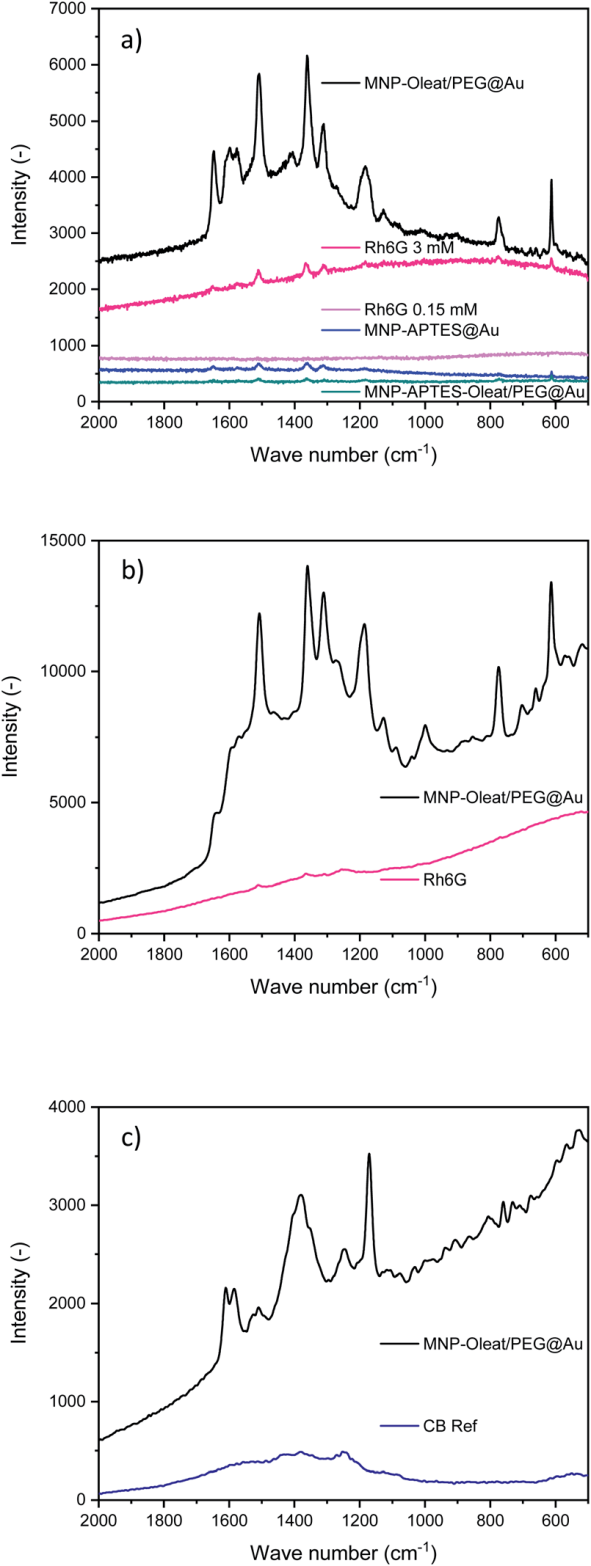
(a) Raman spectra of Rhodamin 6G with and without gold coated nanoparticles excited with a 633 nm laser. (b) Raman spectra of Rhodamin 6G with and without gold coated nanoparticles excited with a 785 nm laser. (c) Raman spectra of Coomassie Blue with and without gold coated nanoparticles excited with a 785 nm laser.

Gold nanoparticles (∼30–40 nm) with an absorbance maximum at 570 nm have been synthesized and used as a reference for the SERS activity of the here synthesized hybrid particles (Fig. S5†). The properties of the synthesized hybrid nanoparticles were tested to enhance Raman scattering. Therefore, the nanomaterials were incubated with the dye Rhodamin 6G (Rh6G) and excited with a 633 nm laser. We were not able to observe any Raman scattering with the solely APTES- and gold-coated nanoparticles which might be due to their small gold particle size. However, both other gold-coated nanoparticle systems demonstrate a SERS effect for Rhodamin 6G with a 633 nm laser (Fig. 5). While the APTES-coated, oleate- and PEG-stabilized system only shows an enhancing effect of a factor of 10–20, the oleate- and PEG-stabilized gold-coated particles demonstrate an enhancement factor of 200 (Fig. 5). These enhancement factors are similar to the findings of Yap *et al.*, who investigated antibody binding events with bifunctional gold-iron oxide nanoparticle systems and a 633 nm laser.^47,60^ Here, the spectrum of dissolved Rhodamin 6G is clearly visible (Fig. 5). The peaks at 1640 cm^−1^ corresponding to C–H bending vibrations are visible as well as the vibrations at 1577 cm^−1^ and 1360 cm^−1^ corresponding to N–H and C–H bending, respectively.^61^

Such an enhancement factor combined with the concentration due to magnetic separation is the ideal fundamental for signalling applications of different molecules. The enhancement factor is significantly stronger than for the hybrid maghemite-gold nanoparticles and the bare gold nanoparticles (Fig. S10–S13†).

In addition to the SERS effect at 633 nm, we tested a 785 nm laser for the detection of Rhodamin 6G and Coomassie Blue (CB) dyes with the gold-coated nanoparticles, that are oleate- and PEG-stabilized.

For Rhodamin 6G the same enhancing effect is visible with a 785 nm excitation as for the 633 nm excitation (Fig. 5b). In the SERS spectra, the vibrations corresponding to Rhodamin 6G are visible. There is a slight change of intensities for different vibration modes. The band corresponding to C–H bending at 1577 cm^−1^ is only visible as a shoulder in the 785 nm Raman spectrum, while the peak at 1185 cm^−1^ corresponding to C–H bending vibrations is even more prominent.^61^

For Coomassie Blue, an enhancing effect similar to the effect of Rhodamin 6G is visible. The bands corresponding to the vibrations at 1538 and 1335 cm^−1^ corresponding to ring vibrations are visible.^60^ Furthermore, the band at 1400 cm^−1^ and the band at 1150 cm^−1^ corresponding to C–H bending vibrations are visible.^61^ The enhancement effect is similar for both dyes and for both laser excitation wavelengths. Here, the enhancement factor is again significantly stronger than for the maghemite gold hybrid materials even though an enhancement effect is also visible for them (Fig. S11 and S13†). The SERS experiments clearly demonstrate the effect of different surface stabilization methods on the performance of a hybrid magnetic gold nanomaterial. It is especially interesting to observe the beneficial effect of PEG as a stabilizing material.^48^ Furthermore, not only homogeneous gold layers or narrowly size-distributed gold hybrid materials can lead to a beneficial SERS effect but also differently-sized and shaped gold iron oxide hybrids. This very well agrees with the spiky and asymmetrically shaped gold particles leading to enhanced Raman scattering.^18,25^

## Conclusions

Hybrid nanoparticles based on magnetic and gold nanomaterials are of great interest and might influence the analytics of tomorrow.^62^ However, especially the sensing properties of gold and how they are affected by the material structure need to be investigated.^63^ To demonstrate the effect of colloidal stability for the preparation of hybrid gold-iron oxide nanomaterials, we applied here different stabilization strategies of the magnetic suspensions for the seeded growth of gold onto their surface and analyzed the SERS effect. Oleate was found to be the best stabilizing agent for narrow suspension size distribution and small mean sizes. Moreover, PEG is a beneficial agent for the homogeneous distribution of gold seeds and the growth of larger particles, that led to an improvement of SERS signals. A significant enhancement effect of the signals of the dyes Rhodamin 6G and Coomassie Blue has been demonstrated for the laser wavelengths 633 and 785 nm. The strategy of coupling gold and iron oxide nanoparticles is an interesting approach for novel signalling applications, which is not only limited to dye detection but also has great potential for molecule detection and identification. Here, not only biosensing for viruses and bacteria are of interest but also establishing completely new ways of enriching and identifying materials.

## Author contributions

Sebastian P. Schwaminger: conceptualization, writing – original draft, validation, visualization, writing – original draft, and writing – review & editing. David Bauer: data curation, formal analysis, investigation, methodology, conceptualization, and visualization. Paula Fraga-García: conceptualization, investigation, methodology, writing – original draft, and writing – review & editing.

## Conflicts of interest

There are no conflicts to declare.

## Supplementary Material

NA-003-D1NA00455G-s001
