# Supplementary material for: Gold-iron oxide nanohybrids: insights into colloidal stability and surface-enhanced Raman detection
Source: Nanoscale Adv. 2021 Sep 8;3(22):6438–45. doi: 10.1039/d1na00455g (PMC9416941; doi:10.1039/d1na00455g)
Supplement: NA-003-D1NA00455G-s001 [file NA-003-D1NA00455G-s001.pdf]

Electronic Supplementary Material (ESI) for Nanoscale Advances.
This journal is © The Royal Society of Chemistry 2021

1 Electronic Supplementary Material

Gold-iron oxide nanohybrids: Insights into colloidal stability and surface enhanced Raman detection

Sebastian P. Schwaminger,[a,*] David Bauer[a] and Paula Fraga-García,[a,*]

[a]Bioseparation Engineering Group, Department of Mechanical Engineering, Technical University of Munich, Garching, Germany

Synthesis of maghemite nanoparticles and gold-iron oxide nanohybrids

Oxidation of magnetite by heating under air flow in a laboratory glass reactor[1]: 1 L of a suspension of freshly prepared Fe_3O_4 nanoparticles with a concentration of 7 g L^{-1} is heated to 60 °C under stirring at 350 rpm. After maintaining the temperature stable for 10 min air is blown into the reaction mixture by raising the stirring speed to 400 rpm. Aliquots of the reaction mixture are taken after 0, 15, 45 min and 2, 4 and 24 h and most of the material is frozen at – 80 °C.[1]

Stabilization with TMAOH[2]: Treatment of particles with tetramethylammonium hydroxide (TMAOH) was carried out by mixing a particle suspension containing 40 mg with 10 mL of 0.1 M TMAOH, separating the particles by centrifugation (3500 rpm, 15 min) or magnetic separation (for minimum of 24 h) and resuspending the particles in the same amount of TMAOH again. This procedure was carried out three times before using the particles in the gold coating experiments. The final precipitate was resuspended in 10 mL of deionised H_2O and sonicated for three minutes at 25% amplitude.

Au seeded growth on maghemite nanoparticles[2]: The particles were adjusted to a concentration of 0.1 g L^{-1} and a pH of 12 with NaOH. The mixture was sonicated prior to the addition of sodium citrate (2.5 mL, 0.1 M) and stirred for 5 min. Addition of 4 mL hydroxylamine hydrochloride $NH_2OH(HCl)$ (0.2 M) and 9.0 mL $HAuCl_4$ (29 mM) succeeded in 10 iteration steps . After the last iteration the mixture was kept stirring for another 30 minutes, before washing it three times with deionised water.

Synthesis of gold nanoparticles

Non spherical gold nanoparticles were synthesized according to a procedure by Minati et al.[3] In a glass vial, 3 mL of $NH_2OH \cdot HCl$ (0.1M, pH = 12 adjusted by NaOH/HCl) were stirred with a magnetic stirring bar at 700 rpm at room temperature. Addition of $HAuCl_4$ (0.01 M, 300 µL) led to immediate colour change and the solution became blue. After stirring it for 2 more minutes the suspension was washed three times by centrifugation (3 min, 15000 g), replacing the supernatant with 4 mL deionised H_2O and dispersing it again by ultrasound treatment.

Characterization methods

Crystal structure and phase purity of the precipitates were examined at room temperature via powder X-ray diffraction (XRD) from the lyophilized samples. The Stadi-P X-Ray diffractometer employed in this work is equipped with a molybdenum X-Ray source (Ge(111) monochromator, Kα1 radiation, λ = 0.7093 Å) and a Mythen 1K detector.

Scanning electron microscopy (SEM) and energy dispersive X-ray spectrocopy (EDX) was conducted with a JEOL JSM-7500F instrument.

Simultaneous thermal analysis (STA): Thermogravimetric measurements of the plane and APTES functionalized MNPs were carried out from lyophilized samples by the simultaneous thermal analysis system STA 449C Jupiter (Netzsch Gerätebau GmbH, Germany). The weight loss and the heat transfer were recorded at a heat rate of 10 K min^{-1} (303 K – 1373 K) under nitrogen atmosphere.

Table S1 Synthesis conditions of hybrid particles and size characteristics derived from TEM, DLS and XRD. TEM size refers to the primary particle diameter size (the diameter of one single nanocrystal). The DLS diameter refers to the hydrodynamic diameter of the stable particle aggregates. The Scherrer diameter refers to the diameter of gold nanoparticles and is obtained from the FWHM of the reflexes corresponding to gold <111>.

**surface treatment**	**$V_{citrate}$ (0.1 M)**	**c_{react} (MNP) [g/L]**	**V_{NH2OH} (0.2 M) [mL]**	**V_{HAuCl4} (29 mM) [mL]**	**m_{MNP} [mg]**	**TEM size (nm)**	**TEM deviation (nm)**	**DLS diameter (nm)**	**PDI**	**Scherrer diameter (nm)**
γ-Fe_2O_3-TMAOH	2.5	0.5	4.0	9.0	50.0	10.6	3	96.1	0.26	27.4
Fe_3O_4-oleate / PEG	3	0.1	3.0	6.5	45	9.4	2.9	1151.5	0.30	32.2
Fe_3O_4-APTES, oleate/ PEG	3.5	0.1	3.0	6.5	40	10.4	2.8	206.3	0.21	16.8
Fe_3O_4-APTES	2	0.1	3.8	5.3	45	17.9	10.5	187.0	0.10	41.1

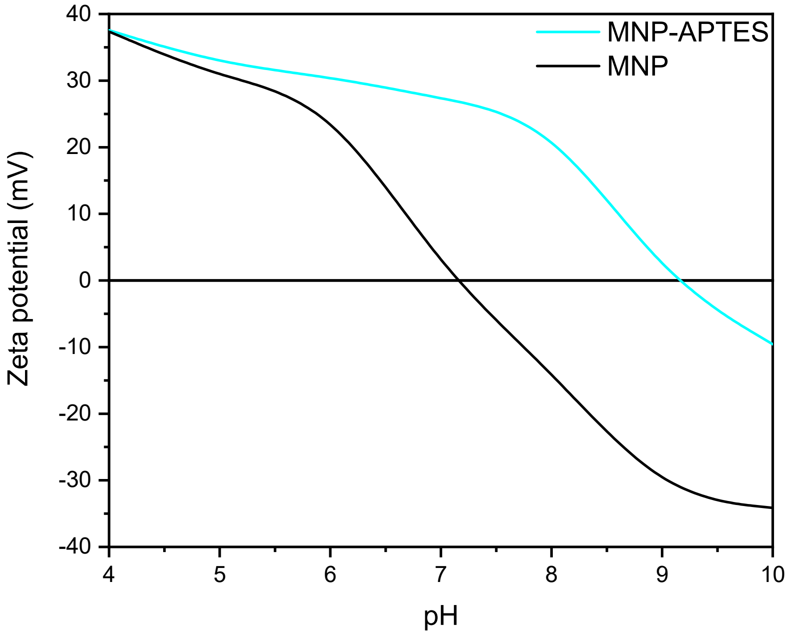

Fig. S1 Zeta potential of magnetite nanoparticles and APTES-coated nanoparticles from pH 4 to 10 in pH steps of 1. HCl and NaOH were used as titrands.

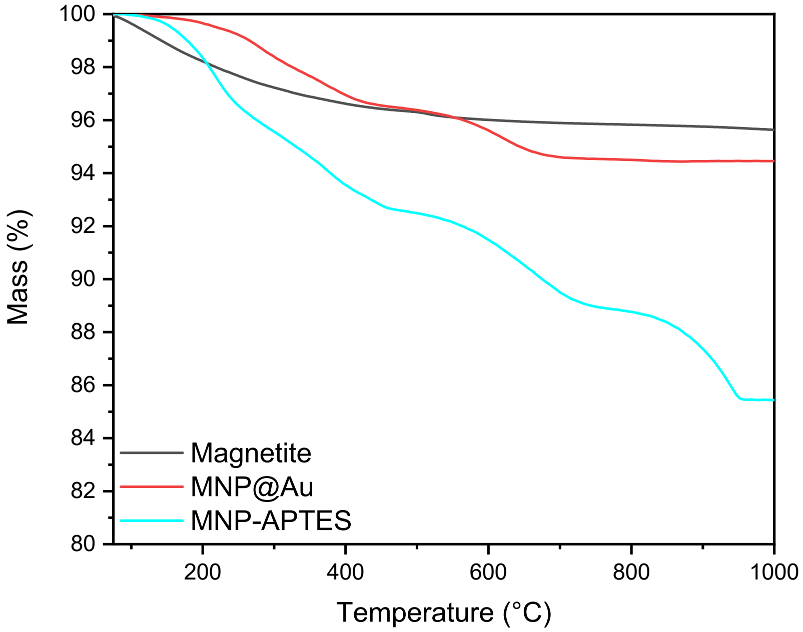

Fig. S2 Thermogravimetry of magnetite nanoparticles, Gold-MNP hybrid particles (stabilised with TMAOH) and MNP-APTES nanoparticles from 75 to 1000 °C with a heating rate of 10 K min^{-1} under nitrogen atmosphere.

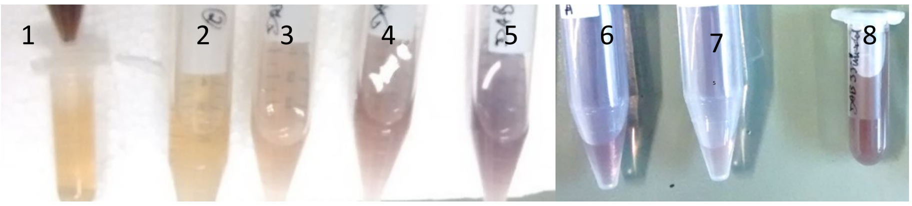

Fig. S3 Images of the aliquots after each addition step from left to right.

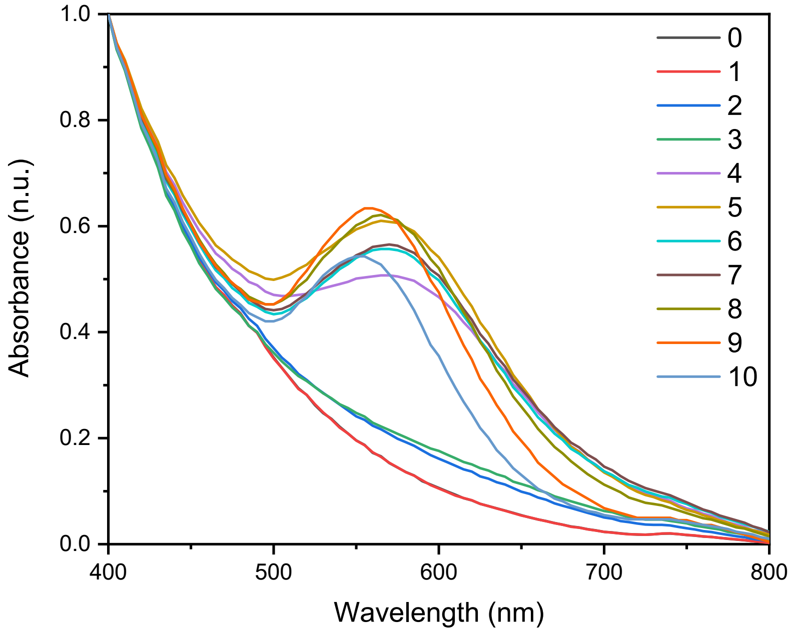

Fig. S4 UV/Vis spectra for multiple steps (1-10) in the gold coating process for TMAOH-stabilised maghemite nanoparticles.

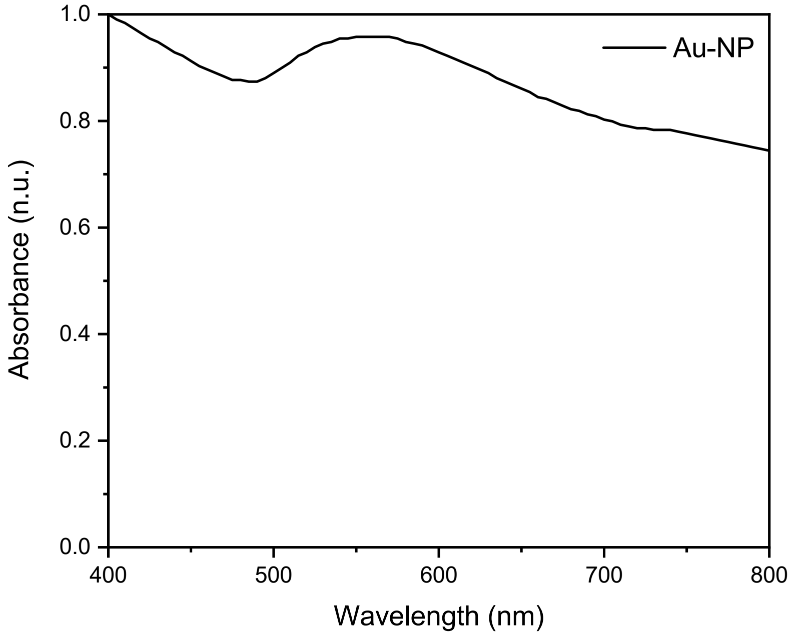

Fig. S5 UV/Vis spectra for gold nanoparticles.

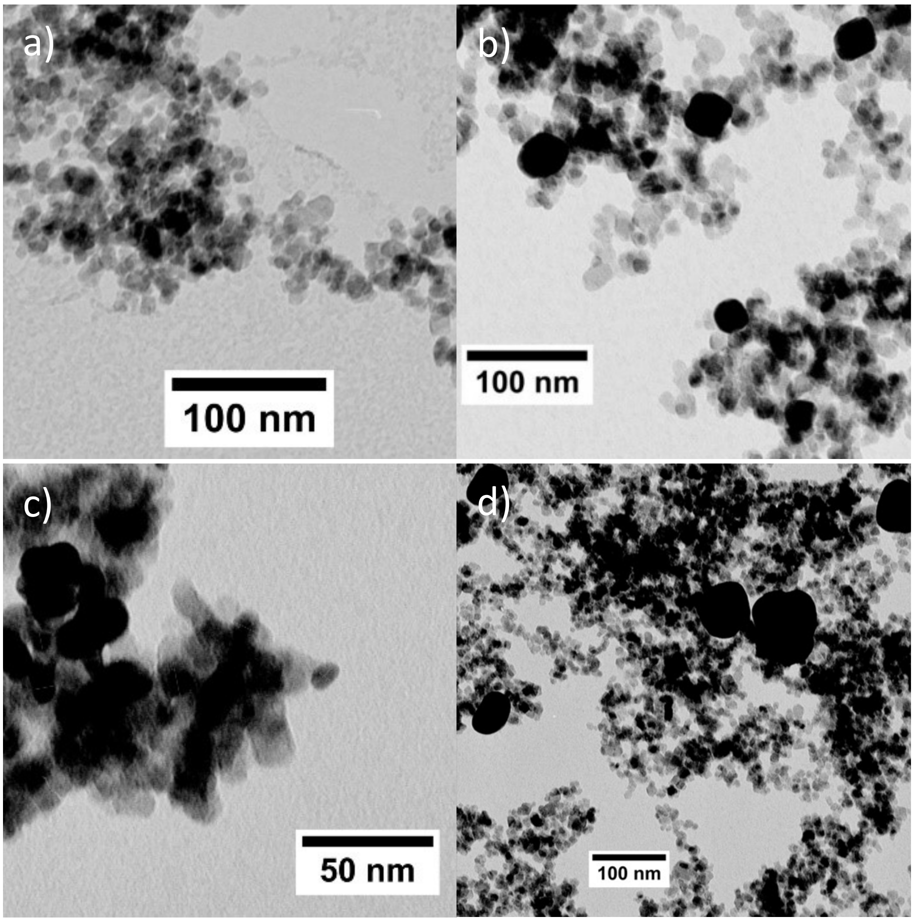

Fig S6 TEM for (a) magnetite nanoparticles, (b) gold coated nanoparticles stabilised with APTES, (c) gold coated nanoparticles stabilised with oleate and PEG and (d) of TMAOH stabilised maghemite nanoparticles.

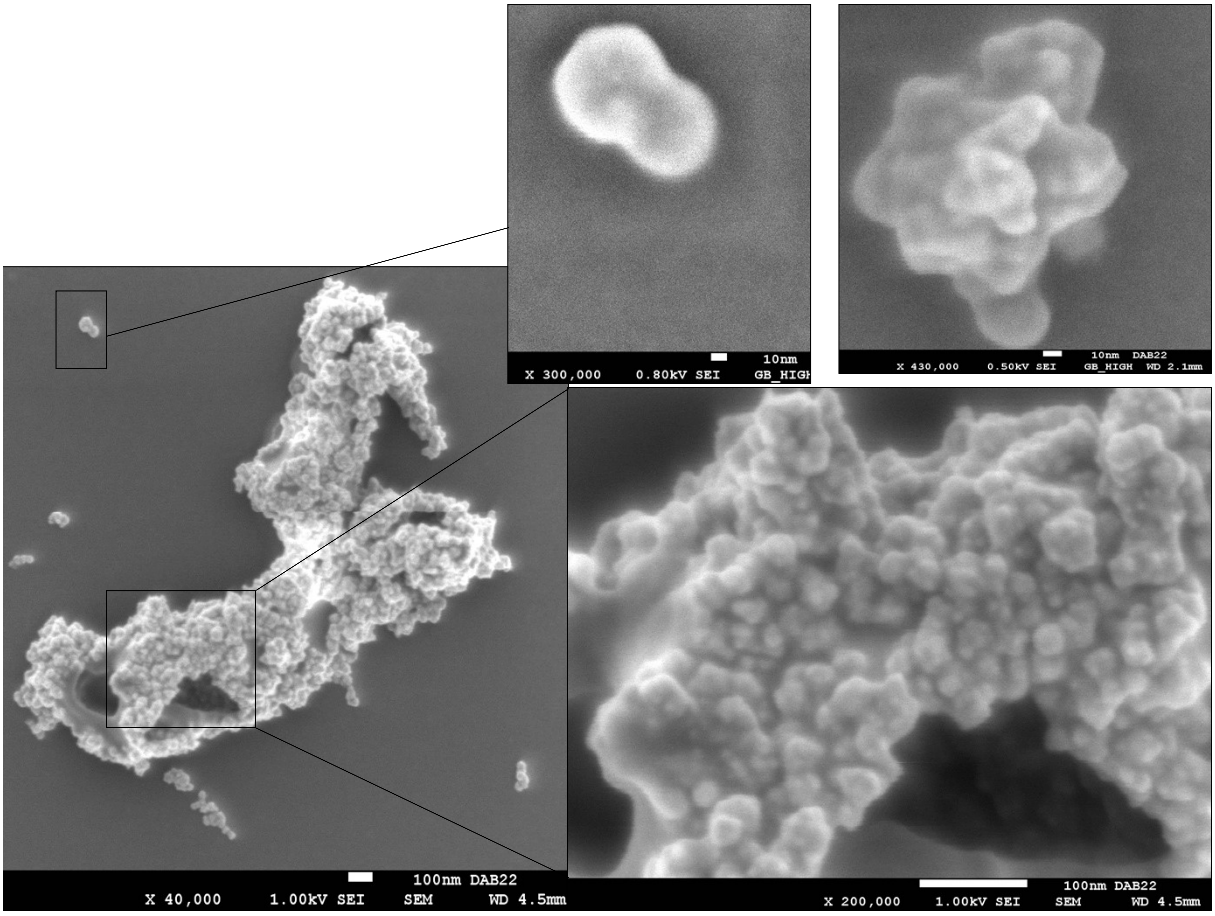

Fig S7 SEM images of gold nanoparticles. Images from Katia Rodewald (Wacker Institute of Macromolecular Chemistry, Technical University of Munich).

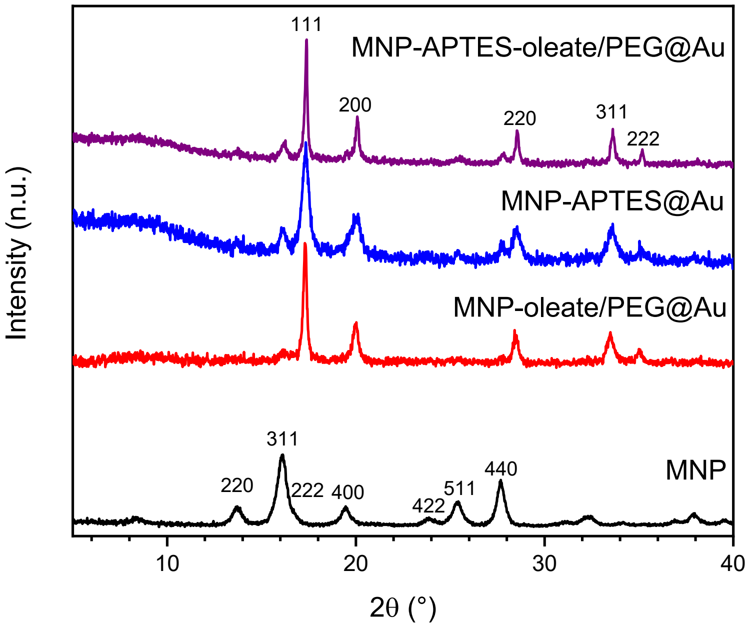

Fig S8 XRD for gold coated nanoparticles stabilised with APTES, APTES, oleate and PEG as well as only stabilised with oleate and PEG. The diffractogram of magnetite nanoparticles (MNP) is added as

a reference from CrystEngComm, 2017, 19, 246-255. DOI: 10.1039/C6CE02421A with permission from the Royal Society of Chemistry.

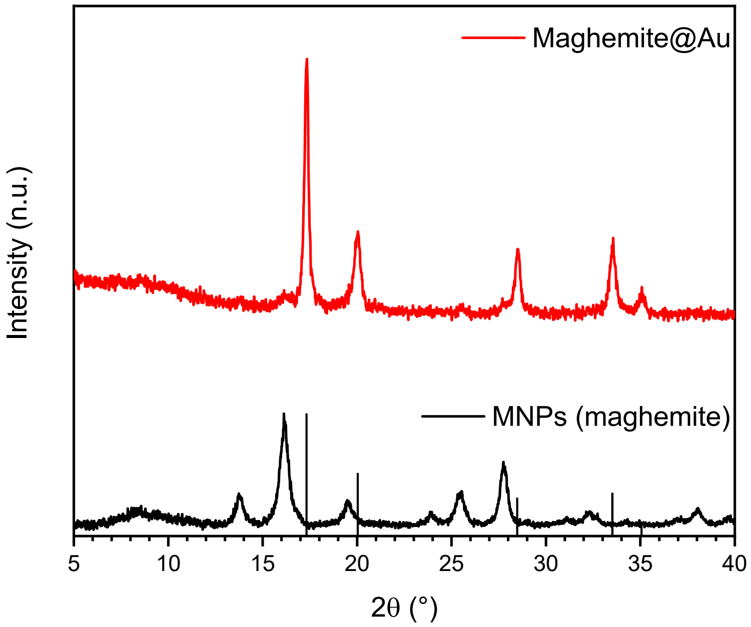

Fig S9 XRD for gold coated nanoparticles stabilised with TMAOH. XRD of maghemite nanoparticles is added as a reference from CrystEngComm, 2017, 19, 246-255. DOI: 10.1039/C6CE02421A with permission from the Royal Society of Chemistry.

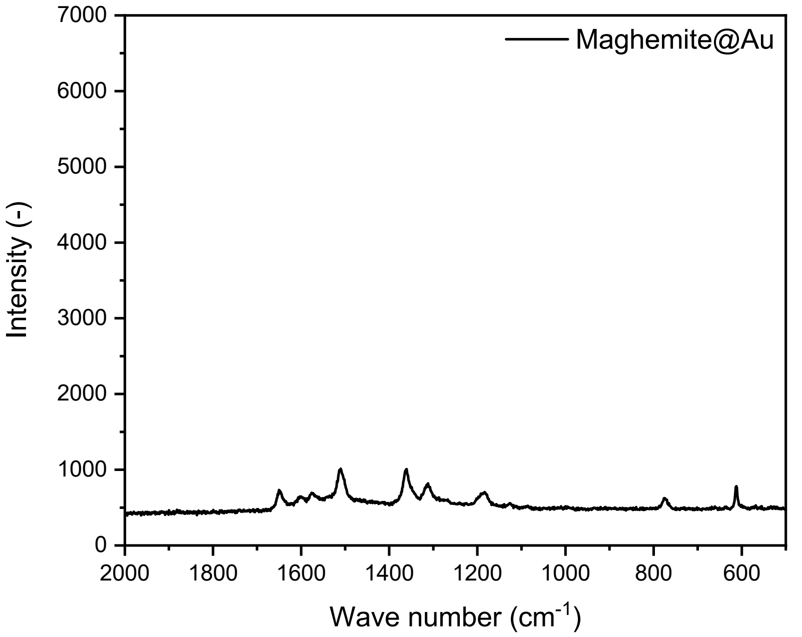

Fig S10 Raman spectrum of Rhodamin 6G with gold coated maghemite nanoparticles (TMAOH stabilized) excited with a 633 nm laser.

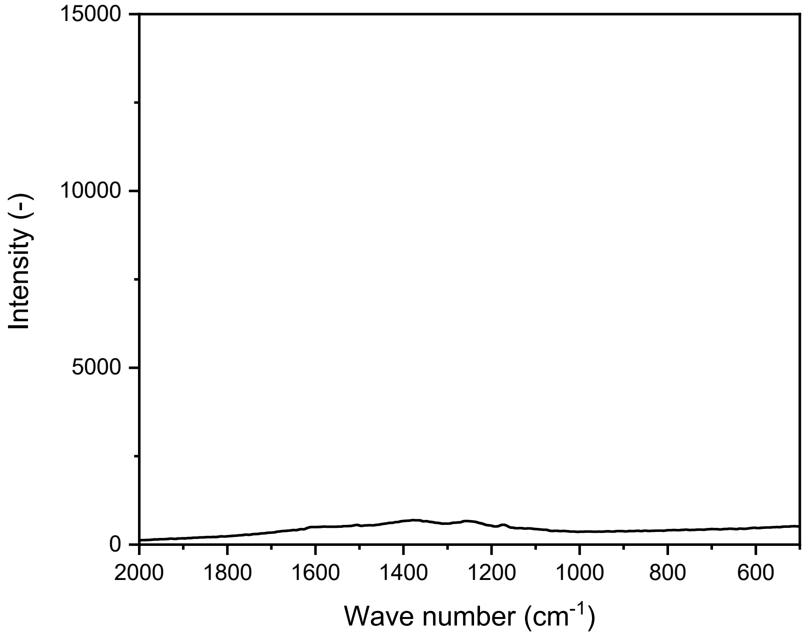

Fig S11 Raman spectrum of Rhodamin 6G with gold coated maghemite nanoparticles (TMAOH stabilized) excited with a 785 nm laser.

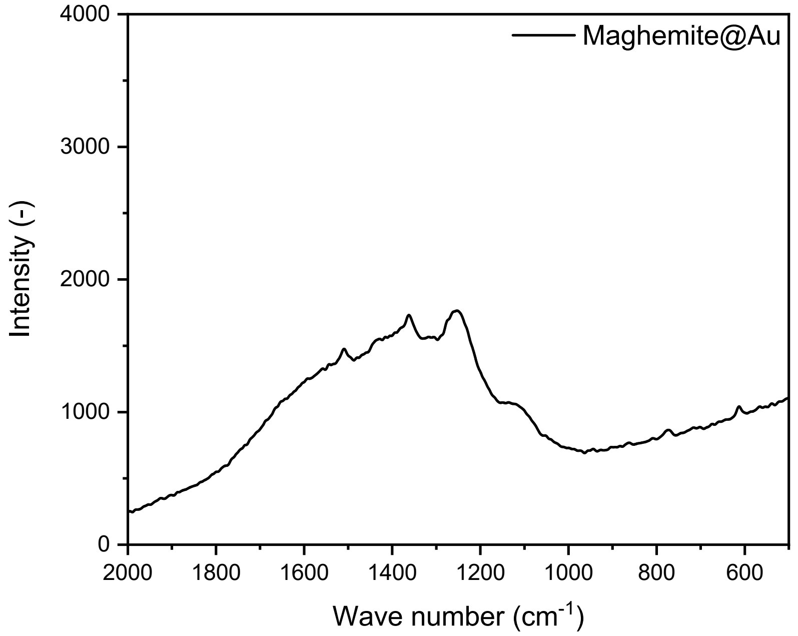

Fig S12 Raman spectrum of Coomassie blue with gold coated maghemite nanoparticles (TMAOH stabilized) excited with a 785 nm laser.

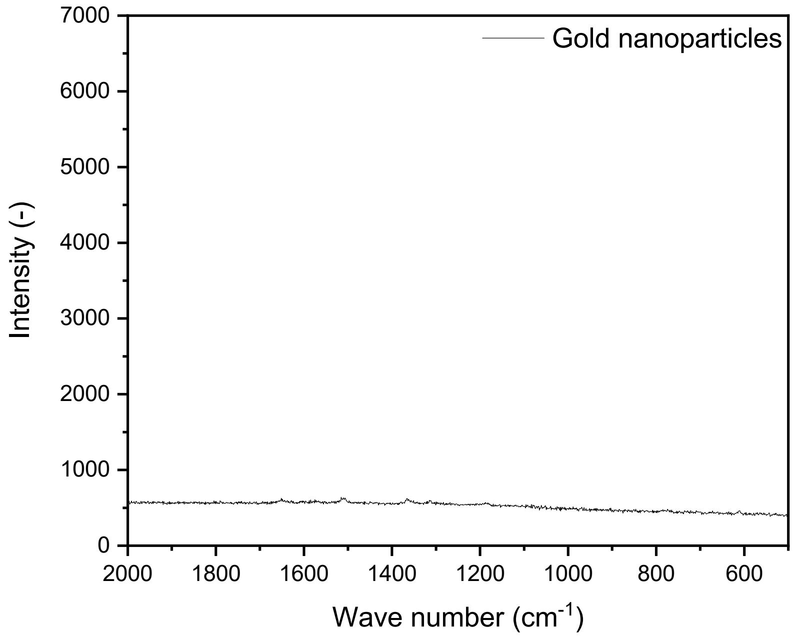

Fig S13 Raman spectrum of Rhodamin 6G with bare gold nanoparticles excited with a 633 nm laser.

References

1 S. P. Schwaminger, D. Bauer, P. Fraga-García, F. E. Wagner and S. Berensmeier, *CrystEngComm*, 2017, **19**, 246–255.

2 J. L. Lyon, D. A. Fleming, M. B. Stone, P. Schiffer and M. E. Williams, *Nano Lett.*, 2004, **4**, 719–723.

3 L. Minati, F. Benetti, A. Chiappini and G. Speranza, *Colloids Surf. A*, 2014, **441**, 623–628.
